# Expression and Regulation of *FGF9* Gene in Chicken Ovarian Follicles and Its Genetic Effect on Laying Traits in Hens

**DOI:** 10.3390/genes16121452

**Published:** 2025-12-04

**Authors:** Yue Wang, Xinmei Shu, Yuanyuan Guo, Qingqing Wei, Yunliang Jiang

**Affiliations:** 1 College of Animal Science and Technology, Shandong Agricultural University, Taian 271017, China; yuewang_224@163.com (Y.W.); idshuxm@126.com (X.S.); guoyy2026@163.com (Y.G.); weiqq@sdau.edu.cn (Q.W.); 2 Shandong Provincial Key Laboratory for Livestock Germplasm Innovation & Utilization, Shandong Agricultural University, Taian 271017, China

**Keywords:** chicken, FGF9, follicle, expression, egg-laying traits, SNP

## Abstract

Objectives: Fibroblast growth factor 9 (FGF9), a crucial member of the FGF family, functions as an intercellular signaling molecule involved in angiogenesis, embryogenesis, and tissue repair. Our previous study demonstrated that *FGF9* expression in chicken hierarchical granulosa cells (Post-GCs) is regulated by LSD1 Ser54 phosphorylation and that FGF9 promotes cell proliferation. This study aims to analyze the expression and regulation of the *FGF9* gene in chicken ovarian follicles and its genetic effect on laying traits in hens. Methods: Chicken *FGF9* mRNA expression patterns were examined by real-time quantitative PCR (RT-qPCR). Detection of single nucleotide polymorphisms (SNPs) was performed using PCR amplification and Sanger sequencing. Transcription activity was compared using dual-luciferase reporter assay. Results: Following follicle selection, chicken *FGF9* expression significantly decreased in granulosa cells (*p* < 0.05) while it increased in theca cells (*p* < 0.05). Hormonal treatments revealed differential regulation; estradiol and FSH downregulated *FGF9* in both pre-hierarchical and hierarchical granulosa cells (*p* < 0.05), whereas progesterone exhibited opposing effects, suppressing expression in pre-hierarchical granulosa cells (Pre-GCs) but stimulating its expression in Post-GCs (*p* < 0.05). In theca cells, estradiol consistently inhibited *FGF9* expression (*p* < 0.05), while FSH only affected *FGF9* expression in pre-hierarchical follicles. Six SNPs in the promoter region (g.−1965G>A, g.−2177G>A, g.−2289G>A, g.−3669A>G, g.−3770A>G, g.−3906G>A) were identified, five of which (g.−1965G>A, g.−2177G>A, g.−2289G>A, g.−3669A>G, g.−3906G>A) showed significant associations with egg production traits. Notably, alleles A (g.−2289), G (g.−3669), and A (g.−3906) enhanced the transcription activity of chicken *FGF9* in Pre-GCs. Conclusions: These findings provide novel insights into the expression pattern and regulatory mechanisms of chicken *FGF9* during follicular development and identify some genetic markers for egg-laying traits in chickens.

## 1. Introduction

Fibroblast growth factors (FGFs) represent a family of potent growth factors with significant roles in tissue repair and regeneration, including at least 20 members, with each comprising approximately 150–200 amino acids. Among them, FGF9 is a 208-amino acid protein first isolated from human glioma cell cultures in 1993 [1,2] and exhibits remarkable evolutionary conservation of >93% homology across mice, rats, and humans. FGF9 is expressed in many tissues and plays various roles through autocrine/paracrine mechanisms [3,4]. In the mouse XY gonad, Sry promotes the expression of Fgf9 while inhibiting Wnt4, thereby directing the development of the testis pathway, suggesting that FGF9 and WNT4 play opposing roles in the regulation of mammalian sex determination [5]. FGF9 is also reported to be involved in ovarian follicle development in several mammalian species. The expression of *FGF9* in the murine ovary stimulates progesterone production in granulosa cells [6]. In cattle, FGF9 acts as an anti-differentiation factor [7,8] by promoting granulosa/theca cell proliferation while inhibiting steroidogenesis via cAMP pathway modulation [9,10] and in pigs, in synergy with IGF-1, FGF9 enhances estradiol production and follicular development [11,12].

In chicks, *FGF* expression in the developing mesonephros plays a role in gonad development during the sexually indifferent stages by promoting gonadal cell proliferation and the expression of gonadal marker genes [13]. In hens, ovarian follicle development occurs in a well-defined hierarchical sequence, beginning with pre-hierarchical stages, which include the small white follicle (<2 mm, SW), large white follicle (2–5 mm, LW), and small yellow follicle (6–8 mm, SY). This is followed by the development of hierarchical follicles, ranging from F6 to F1 [14,15]. This precisely regulated process involves complex endocrine/paracrine interactions that are regulated by sex hormones and other cellular factors [16]. Our previous study demonstrated that *FGF9* expression was reduced in hierarchical granulosa cells (Post-GCs) following follicle selection. Moreover, FGF9 was found to promote the proliferation of granulosa cells and enhance the mRNA expression of hydroxy-delta-5-steroid dehydrogenase, 3 beta- and steroid delta-isomerase 1 (*Hsd3b*), and steroidogenic acute regulatory protein (*StAR*) [17]. These findings suggest that FGF9 plays a critical role in the development of chicken follicles. Single-nucleotide polymorphisms (SNPs), the most abundant genetic variations (90% of all DNA polymorphisms), show particularly high frequency in chickens, approximately 6–7 times greater than in humans [18]. This study first analyzed the expression of *FGF9* mRNA in ovarian follicles of different sizes, as well as in follicular granulosa cells and theca cells, then examined the effect of estradiol, FSH, and progesterone on *FGF9* mRNA expression in follicular granulosa and theca cells, and finally analyzed SNPs in the promoter region of chicken *FGF9*, which are associated with egg-laying traits.

## 2. Materials and Methods

### 2.1. Animals and Sample Collections

In this study, ovarian follicles were collected from regularly laying Hy-Line Brown hens, aged 30 weeks. When collecting samples, 40 Hy-Line Brown hens were randomly selected and sacrificed by decapitation in compliance with the requirements of animal welfare of Shandong Agricultural University (No. SDAUA-2022-36). Pre-hierarchical and hierarchical follicles were separately collected and immediately preserved in liquid nitrogen to extract total RNA or used to prepare primary theca and granulosa cells. Each experiment included three technical replicates, with measurements taken from multiple pools of follicles, where each pool comprised follicles from three hens.

For polymorphism analysis, 399 Jining Bairi hens, 40 Jinghong laying hens, 40 Zaozhuang Sunzhi hens, and 40 Langya hens were sampled. A sample of approximately 2 mL of blood from each hen was obtained from the wing vein. Genomic DNA from blood was extracted using a DNA extraction kit (DP304, TIANGEN, Beijing, China). A subset of 399 Jining Bairi hens with egg-laying records was chosen for the association analysis between *FGF9* genotypes and egg-laying traits. Production traits, including age at first egg (AFE), body weight at first laying (BW), egg number at 52 weeks (E52), egg weight at first laying (EW), and largest clutch size (LCS), were recorded for each hen.

### 2.2. Cell Culture and Treatment

Follicles were divided into pre-hierarchical and hierarchical follicles. The egg yolk was carefully squeezed from the follicles with tweezers and washed with phosphate-buffered saline. Pre-hierarchical follicles, including SW, LW, and SY, were treated with collagenase II (Coolaber, Beijing, China) at 37 °C for 5 min to dissociate granulosa cells (Pre-GCs), followed by an additional 30 min to disperse theca cells (Pre-TCs). For hierarchical follicles, the granulosa cell layers were dissociated using 0.25% trypsin-EDTA (Gibco, Grand Island, NY, USA) for 10 min to prepare hierarchical granulosa cells (Post-GCs), while theca cell layers were treated with collagenase II for 30 min to prepare hierarchical theca cells (Post-TCs). All isolated cells were filtered through a 200-mesh sieve, suspended in M199 medium (Gibco, Grand Island, NY, USA) supplemented with 5% fetal bovine serum (FBS; VivaCell, Shanghai, China) and 1% penicillin/streptomycin (Solarbio, Beijing, China), then seeded in 24-well culture plates. The cells were cultured in a humidified atmosphere with 5% CO_2_ at 39 °C. After 24 h, the cells were exposed to varying concentrations of estradiol (E2; Sigma-Aldrich, St. Louis, MO, USA), progesterone (P4; Sigma-Aldrich, St. Louis, MO, USA), or FSH (NOVUS, Centennial, CO, USA), and cultured in M199 medium for an additional 24 h.

### 2.3. RNA Extraction and Real-Time Quantitative PCR (RT-qPCR)

Total RNA was extracted from follicular tissues and follicular cells using an RNA Simple Total RNA Extraction Kit (TIANGEN, Beijing, China). The cDNA synthesis was carried out using the Evo MMLV RT Mix Kit with gDNA Clean for qPCR (Accurate Biotechnology, Changsha, China). The RT-qPCR reaction system consisted of 10 μL of 2× SYBR Green Pro Taq HS Premix (Accurate Biotechnology, Changsha, China), 0.4 μL each of FGF9-F and FGF9-R primers (Table 1), 2 μL of cDNA, and 7.2 μL of ddH_2_O. The reaction conditions for RT-qPCR were 95 °C for 30 s, 95 °C for 5 s, and 62 °C for 30 s, for 40 cycles. The reaction conditions for the melting curve were 95 °C for 10 s, 65 °C for 60 s, and 97 °C for 1 s. The expression of related genes was calculated using the 2^−ΔΔCt^ method using *GAPDH* as the reference gene. 

### 2.4. Construction and Dual-Luciferase Analysis of Chicken FGF9 Promoter Vectors

The promoter region of the chicken *FGF9* gene, designated as F1, was amplified from −3127 bp to +514 bp (with the transcription start site defined as +1) using primers F1-KpnI and R-MluI (Table 1). The upstream primer included a *Kpn*I recognition site and protected bases at the 5′ end, while the downstream primer incorporated a *Mlu*I recognition site and protected bases at the 5′ end. The amplification products were purified and recovered after gel electrophoresis, followed by a double restriction enzyme digestion of the purified fragments. The digested products were again purified after gel electrophoresis and ligated to the enzyme-digested and purified pGL3-Basic vector using Ligation Solution A (Accurate Biotechnology, Changsha, China). Using F1 as the template, primers were designed to generate deletion constructs of varying lengths within the promoter region. While keeping the downstream primer unchanged, upstream primers were designed starting from the 5′ end, spaced approximately 600 bp apart. The upstream primers F2-KpnI, F3-KpnI, F4-KpnI, F5-KpnI, and F6-KpnI were paired with the downstream primer R-MluI to amplify different-sized promoter fragments. The deletion constructs were generated using the same method as F1 and were designated F2, F3, F4, F5, and F6, respectively. Five mutant vectors were amplified through PCR amplification with specific primer pairs Mut-F-1965/Mut-R-1965 for pGL3-(−1965A), Mut-F-2177/Mut-R-2177 for pGL3-(−2177A), Mut-F-2289/Mut-R-2289 for pGL3-(−2289A), Mut-F-3669/Mut-R-3669 for pGL3-(−3669G), and Mut-F-3906/Mut-R-3906 for pGL3-(−3906A) (Table 1), using F1 as a template.

pGL3-Basic, F1-F6, and each of the mutant vectors were transfected into Pre-GCs according to the instructions of the Lipofectamine™ LTX Reagent kit (Thermo Fisher Scientific, Waltham, MA, USA). At least 3 replications of each vector plasmid were used, and 3 independent experiments were conducted. Twenty-four hours later, dual-luciferase activity was measured using the Dual-Luciferase Reporter Assay System (Promega, Madison, WI, USA). Luciferase activity was quantified using a luminometer (Modulus™; Turner Biosystems, Sunnyvale, CA, USA). The ratio of Firefly luciferase activity to Renilla luciferase activity was used to calculate the relative enzymatic activity.

### 2.5. Genotyping

For this study, genomic DNA samples were randomly selected from 34 individuals representing the four chicken breeds of Zaozhuang Sunzhi chicken, Langya chicken, Jinghong layer, and Hy-Line Brown layer. The promoter region of the chicken *FGF9* gene was amplified using primer pairs P-FGF9-F1/R1 and P-FGF9-F2/R2 (Table 1) at a volume of 25 μL. The amplification products were sequenced using Sanger sequencing and analyzed with DNAMAN 7.0 and Chromas Pro 2.6.5 software to determine the genotypes at six sites: g.−1965G>A, g.−2177G>A, g.−2289G>A, g.−3669T>C, g.−3770A>G, and g.−3906G>A.

### 2.6. Statistical Analysis

All data were presented as mean ± standard error and were tested for normality. Comparisons between two groups were performed using the independent Student’s *t*-test, while differences among multiple groups were assessed by one-way ANOVA followed by the LSD multiple comparison test using SPSS 22.0, and *p* < 0.05 was considered statistically significant.

## 3. Results

### 3.1. Expression of Chicken FGF9 mRNA in Different Ovarian Follicles, Follicular Granulosa Cells, and Theca Cells

**Figure 1:**
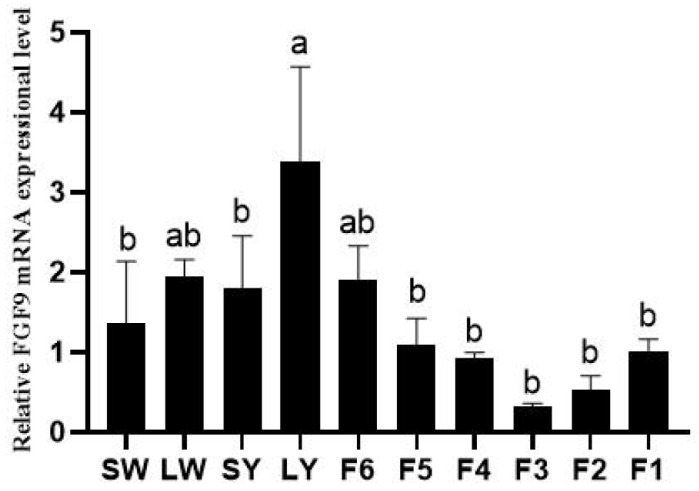
The expression of *FGF9* mRNA in chicken ovarian follicles at different developmental stages (*n* = 3). SW, LW, SY, and LY represent small white follicles, large white follicles, small yellow follicles, and large yellow follicles. F6 to F1 represent hierarchical follicles. Differences in significance are indicated by different lowercase letters (*p* < 0.05).

**Figure 2:**
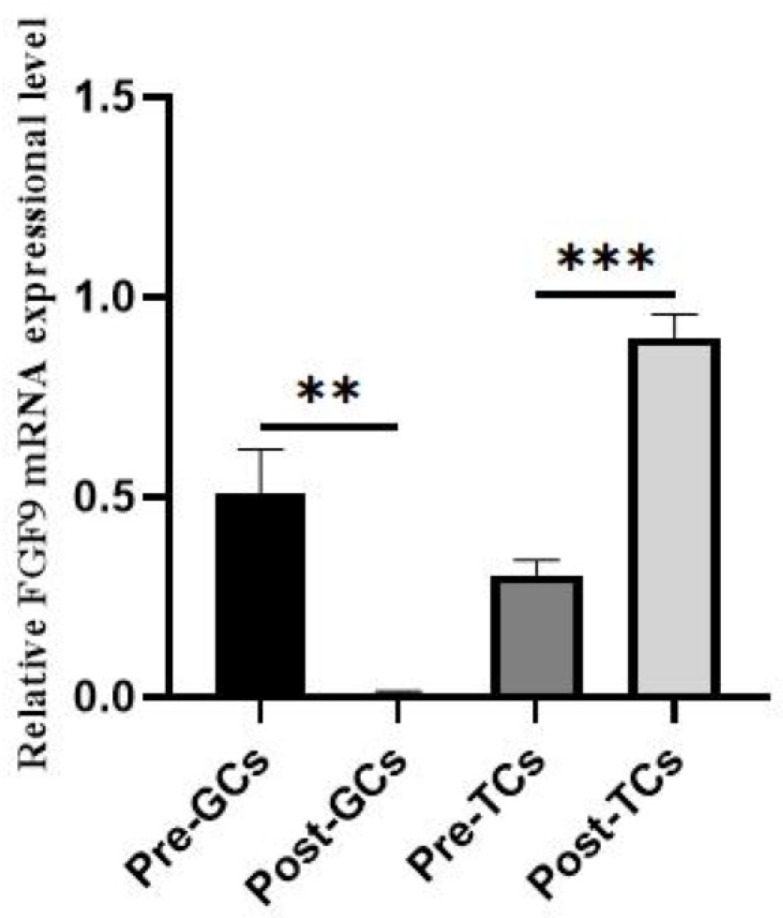
The expression of *FGF9* mRNA in chicken follicular granulosa and theca cells (*n* = 3). Pre-GCs, Post-GCs, Pre-TCs, and Post-TCs represent pre-hierarchical granulosa cells, post-hierarchical granulosa cells, pre-hierarchical theca cells, and post-hierarchical theca cells, respectively. ** *p* < 0.01, *** *p* < 0.001.

Chicken *FGF9* mRNA expression was significantly higher in 9–12 mm follicles (also called large yellow follicles (LY)) than in SW and SY and F5 to F1 hierarchical follicles (*p* < 0.05) (Figure 1). In granulosa cells, *FGF9* mRNA expression significantly decreased in Post-GCs compared to Pre-GCs (*p* < 0.01), while in theca cells, *FGF9* mRNA expression significantly increased in Post-TCs compared to Pre-TCs (*p* < 0.001) (Figure 2).

### 3.2. Effect of Reproductive Hormones on Chicken FGF9 mRNA Expression

**Figure 3:**
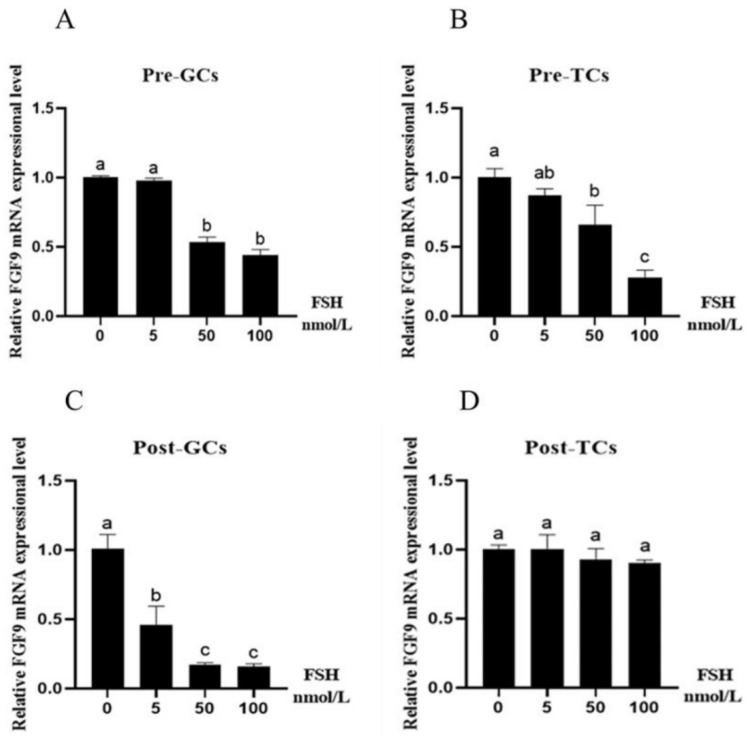
Effect of FSH on *FGF9* mRNA expression in chicken Pre-GCs (**A**), Pre-TCs (**B**), Post-GCs (**C**), and Post-TCs (**D**) (*n* = 3). Pre-GCs, Post-GCs, Pre-TCs, and Post-TCs represent pre-hierarchical granulosa cells, post-hierarchical granulosa cells, pre-hierarchical theca cells, and post-hierarchical theca cells. The expression of *FGF9* was measured by RT-qPCR, with each sample being assayed in triplicate using *GAPDH* as reference and represented as means ± standard error. Differences in significance are indicated by different lowercase letters (*p* < 0.05).

Chicken follicular granulosa cells and theca cells were treated independently with FSH, E2, or P4 at various concentrations for 24 h. Chicken *FGF9* mRNA expression was then quantified by RT-qPCR. At the concentration of 50 nmol/L, FSH significantly reduced the expression of *FGF9* mRNA in Pre-GCs (Figure 3A) and Pre-TCs (*p* < 0.05) (Figure 3B). In Post-GCs, the *FGF9* mRNA expression gradually decreased along with the increase in FSH concentration from 5 nmol/L to 100 nmol/L, and the difference was significant (*p* < 0.05) (Figure 3C); however, the effect of FSH on the expression of *FGF9* mRNA was not different in Post-TCs (Figure 3D).

**Figure 4:**
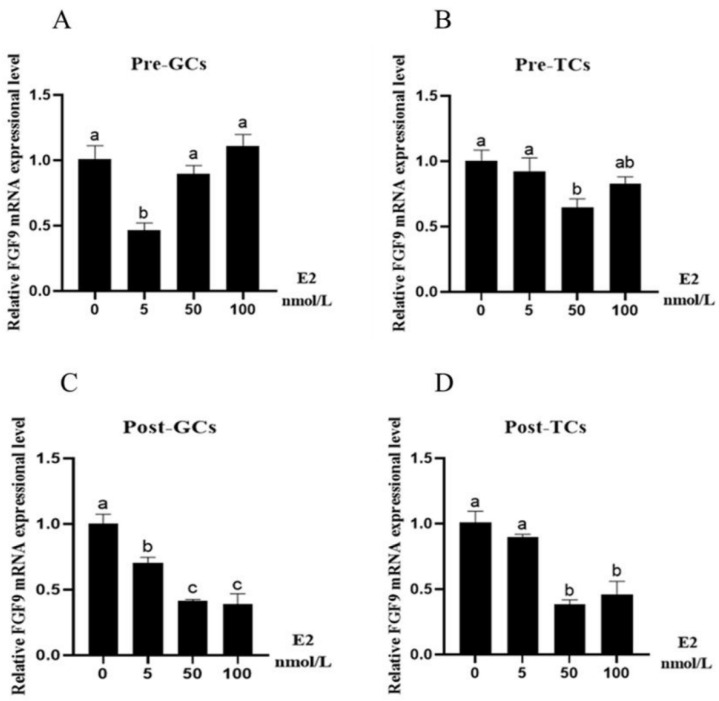
Effect of E2 on *FGF9* mRNA expression in chicken Pre-GCs (**A**), Pre-TCs (**B**), Post-GCs (**C**), and Post-TCs (**D**) (*n* = 3). Pre-GCs, Post-GCs, Pre-TCs, and Post-TCs represent pre-hierarchical granulosa cells, post-hierarchical granulosa cells, pre-hierarchical theca cells, and post-hierarchical theca cells. The expression of *FGF9* was measured by RT-qPCR, with each sample being assayed in triplicate using *GAPDH* as reference and represented as means ± standard error. Differences in significance are indicated by different lowercase letters (*p* < 0.05).

In Pre-GCs, E2 had a significant inhibitory effect on the expression of *FGF9* (*p* < 0.05) at the concentration of 5 nmol/L (Figure 4A), and in Pre-TCs, E2 had a significant inhibitory effect on *FGF9* mRNA expression at the concentration of 50 nmol/L (*p* < 0.05) (Figure 4B). In both Post-GCs and Post-TCs, the expression of *FGF9* was significantly decreased by E2 (*p* < 0.05), from 5 nmol/L to 100 nmol/L(Figure 4C) and from 50 nmol/L to 100 nmol/L (Figure 4D), respectively.

**Figure 5:**
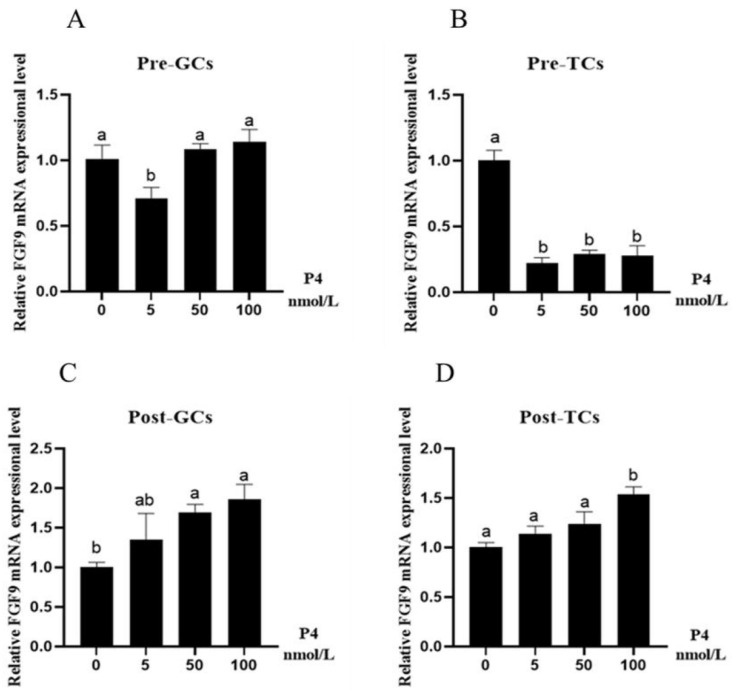
Effect of P4 on *FGF9* mRNA expression in chicken Pre-GCs (**A**), Pre-TCs (**B**), Post-GCs (**C**), and Post-TCs (**D**) (*n* = 3). Pre-GCs, Post-GCs, Pre-TCs, and Post-TCs represent pre-hierarchical granulosa cells, post-hierarchical granulosa cells, pre-hierarchical theca cells, and post-hierarchical theca cells. The expression of *FGF9* was measured by RT-qPCR, with each sample being assayed in triplicate using *GAPDH* as reference and represented as means ± standard error. Differences in significance are indicated by different lowercase letters (*p* < 0.05).

In Pre-GCs, P4 had a significant inhibitory effect on the expression of *FGF9* mRNA (*p* < 0.05) only at the low concentration of 5 nmol/L (Figure 5A), while in Pre-TCs, P4 had a significant inhibitory effect on *FGF9* gene expression (*p* < 0.05) (Figure 5B). In Post-GCs, the expression of *FGF9* was significantly increased at the concentrations of 50 nmol/L and 100 nmol/L of P4 (*p* < 0.05) (Figure 5C), and in Post-TCs, the expression of *FGF9* gradually increased with increasing P4 concentration, and the difference was significant only at the concentration of 100 nmol/L (*p* < 0.05) (Figure 5D).

### 3.3. Promoter Activity of Chicken FGF9 Gene

**Figure 6:**
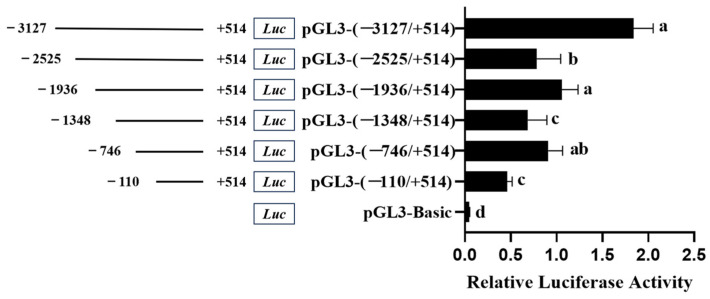
Promoter activity analysis by Dual-Luciferase Reporter Assay System in chicken Pre-GCs. The left is a schematic diagram of different progressive deletions in the 5′ flanking region of the chicken *FGF9* gene; the right shows the activity of the corresponding truncated *FGF9* promoters. Differences in significance are indicated by different lowercase letters (*p* < 0.05).

Chicken Pre-GCs were transfected with deletion vectors with various fragment lengths in the promoter region of the *FGF9* gene, and dual-luciferase activity was measured 24 h later. The results revealed that the dual-luciferase activity was significantly changed after each deletion of the 600 bp fragment from −3127 bp to −110 bp (*p* < 0.05), among which fragments of −3127 bp to −2525 bp, −1936 bp to −1348 bp, and −746 bp to −110 bp habor *cis*-acting positive regulatory elements, while those of −2525 bp to −1936 bp and −1348 bp to −746 bp contain *cis*-acting negative regulatory elements (Figure 6).

### 3.4. Polymorphisms in the Critical Promoter Region of Chicken FGF9 Gene

**Figure 7:**
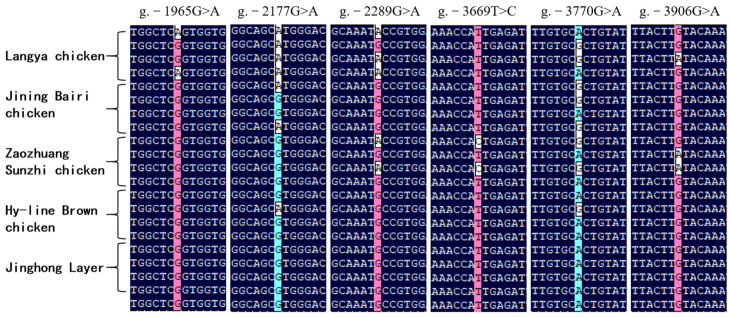
Polymorphisms in the key promoter region of *FGF9* in different chicken breeds. In this experiment, 34 genomic DNAs from each of the five chicken breeds were randomly selected and tested by Sanger sequencing, and the results were analyzed using DNAMAN and Chromas Pro. The figure shows four representative sequencing results for each breed.

**Figure 8:**
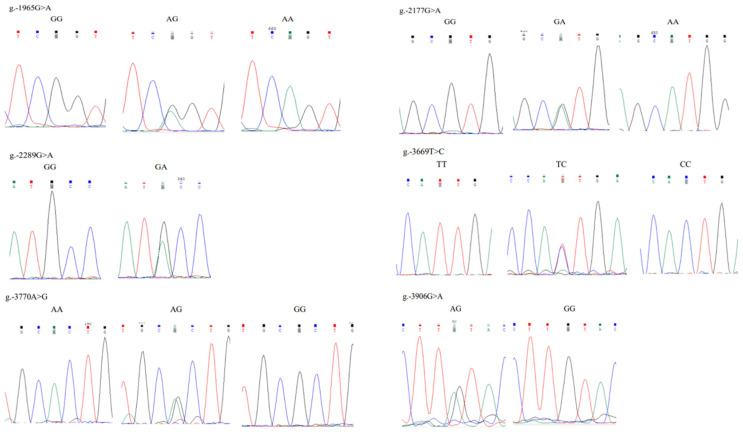
Sanger sequencing results showing polymorphisms at −1965, −2177, −2289, −3669, −3770, and −3906 sites of chicken *FGF9* gene, and six SNPs of g.−1965G>A, g.−2177G>A, g.−2289G>A, g.−3669T>C, g.−3770A>G, and g.−3906G>A were identified, showing two or three genotypes for each SNP.

Sequence alignment of the *FGF9* promoter in five chicken breeds revealed the presence of six SNPs, i.e., g.−1965G>A, g.−2177G>A, g.−2289G>A, g.−3669T>C, g.−3770A>G, g.−3906G>A (Figure 7 and Figure 8).

### 3.5. Association Analysis of SNPs in Chicken FGF9 Promoter Region with Egg-Laying Traits in Jining Bairi Hens

The polymorphic site g.−1965 (G>A) of the chicken *FGF9* gene was significantly associated (*p* < 0.05) with the body weight at first laying (BW); the polymorphic site g.−2177 (G>A) was significantly associated (*p* < 0.05) with the largest clutch size (LCS); the polymorphic site g.−2289 (G>A) was significantly associated (*p* < 0.05) with the age at first egg (AFE); and the polymorphic sites g.−3669 (A>G) and g.−3906 (G>A) were significantly associated (*p* < 0.05) with the number of eggs at 52 weeks (E52) (Table 2).

### 3.6. Effect of SNPs on Transcription Activity of Chicken FGF9

**Figure 9:**
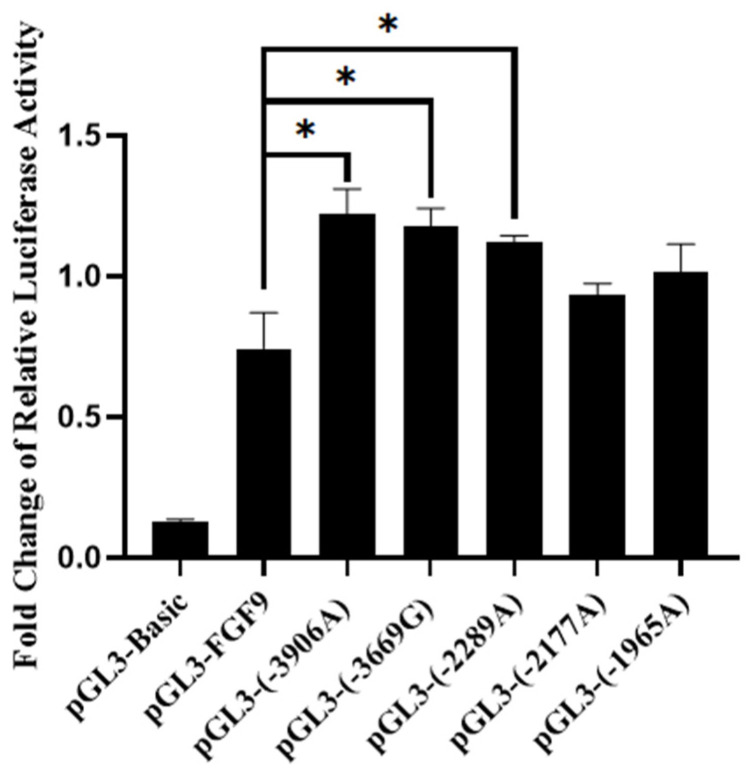
Changes in luciferase activity of vectors pGL3-(−3906A), pGL3-(−3669G), pGL3-(−2289A), pGL3-(−2177A), and pGL3-(−1965A) in chicken Pre-GCs concerning the SNPs g.−3906G >A, g.−3669A>G, g.−2289G >A, g.−2177G >A, and g.−1965G > A compared with pGL3-FGF9 (*n* = 3). * *p* < 0.05.

Chicken Pre-GCs were transfected with the targeted mutation vectors pGL3-(−1965A), pGL3-(−2177A), pGL3-(−2289A), pGL3-(−3669G), and pGL3-(−3906A) and assayed for dual-luciferase activity. The transcription activity of chicken *FGF9* significantly increased after the mutation from G to A at the polymorphic site g.−3906, the mutation from A to G at the polymorphic site g.−3669, and the mutation from G to A at the polymorphic site g.−2289 (*p* < 0.05) (Figure 9).

## 4. Discussion

Follicle selection is a pivotal process in poultry reproduction, directly influencing egg-laying efficiency and egg production traits. Based on our previous study, the mRNA expression dynamics of chicken *FGF9* in different developmental ovarian follicles and in the follicular granulosa and theca cells around follicle selection were analyzed. Then, the effects of FSH, E2, and P4 on *FGF9* mRNA expression were examined in both granulosa cells and theca cells around follicle selection. Finally, five egg production-associated SNPs were identified in the 5′-regulatory region of chicken *FGF9*, three of which affected the transcription activity of *FGF9*. These data suggest that FGF9 is likely involved in chicken follicle selection, and three SNPs of *FGF9* could be used as potential DNA markers for improving egg laying performance in chickens.

In chicken ovarian follicles, *FGF9* mRNA expression exhibits a declining trend after follicle selection, especially in F3 follicles (Figure 1), and was significantly decreased in Post-GCs compared to those of Pre-GCs, which is consistent with our previous study [17]. A study on *FGF9* expression has not been reported in other poultry species, including duck, goose, and quail. Mammalian *FGF9* expression in follicles is mostly reported in cattle. The abundance of bovine *FGF9* mRNA in granulosa cells and theca cells was several-fold greater in small (1–5 mm) than in large follicles (8–22 mm) [19], which is consistent with this study. Additionally, *FGF9* expression increases in both the granulosa and theca cells of medium-sized follicles during the development of dominant follicles and is lower in dominant E2-active follicles compared to subordinate E2-inactive follicles [20]. The expression change in bovine FGF9 in granulosa cells is similar to that in this study, but differs with regard to theca cells, which is likely due to the differences in female reproduction strategy. For example, in cattle, FGF9 promotes post-hierarchical granulosa cell proliferation and steroidogenesis, with FSH upregulating its expression via the cAMP/PKA pathway [9]. Additionally, one study in rats indicates that a deficiency in FGF9 leads to increased follicular atresia, highlighting its essential role in the survival of granulosa cells [6]. The downregulation of *FGF9* in chicken ovarian granulosa cells may reflect species-specific reproductive strategies; as oviparous animals, chickens prioritize rapid follicular differentiation and ovulation over sustained proliferation [21]. In contrast to granulosa cells, *FGF9* mRNA expression in theca cells of chicken ovarian follicles significantly increased (Figure 2). After follicle selection, chicken post-hierarchical theca cells require rapid vascularization to support yolk deposition [22]; the upregulated *FGF9* in Post-TCs may facilitate this process by activating the VEGF pathway, consistent with its pro-angiogenic role in mammals [23]. 

The effects of FSH, E2, and P4 on *FGF9* mRNA expression were further investigated in chicken ovarian follicular granulosa and theca cells. FSH treatment reduced *FGF9* mRNA expression in Pre-GCs, Post-GCs, and Pre-TCs; however, this inhibitory effect is not observed in cattle [10]. E2 treatment reduced *FGF9* mRNA expression in Pre-GCs, Post-GCs, Pre-TCs, and Post-TCs. P4 treatment reduced *FGF9* mRNA expression in Pre-GCs and Pre-TCs, but increased its expression in Post-GCs and Post-TCs. These results suggest that sexual hormones generally suppressed *FGF9* mRNA expression in the granulosa and theca cells; however, after follicle selection, P4 enhanced *FGF9* mRNA expression. The effect of E2 and P4 on *FGF9* mRNA was not reported in cattle or other mammalian species. A similar study is reported on rat ovary and human stromal cells. In immature rat ovaries, *FGF9* mRNA expression levels were significantly reduced following treatment with PMSG for 48 h, hCG for 8 h, or a combined PMSG/hCG (P/h) treatment for 48 and 8 h, respectively. In contrast, the estrogen-induced development of large preantral follicles had no significant effect on *FGF9* expression [6]. In human endometrial stromal cells, the expression of FGF9 is induced by 17β-estradiol but not by progesterone [24]. These data suggest that the effect of reproductive hormones on *FGF9* expression is different between chicken and mammalian species. 

Additionally, the P4-induced upregulation of *FGF9* in Post-GCs and Post-TCs aligns partially with mammalian findings, where P4 inhibits the apoptosis of post-hierarchical granulosa cells [25]. In chickens, P4 also enhances FGF9 expression in Post-TCs, suggesting coordinated roles in post-hierarchical granulosa cell luteinization and post-hierarchical theca cell functional remodeling to maintain the ovulatory microenvironment [26]. Notably, the lack of antral follicles in chickens requires direct cell–cell signaling for follicular selection [27]. Additionally, the dynamics of *FGF9* in specific cell types may indicate its role in regulating bidirectional communication between Post-GCs and Post-TCs. These results revealed the species-specific regulatory network of *FGF9* and suggest potential unique communication mechanisms between granulosa cells and theca cells within the avian ovary, offering novel perspectives for cross-species comparative studies in reproductive biology.

The critical regulatory regions of chicken *FGF9* transcription were further identified in Post-GCs. Five SNPs existing in these regions were found to be associated with egg-laying traits; among them, g.−3906 (G>A), g.−3669 (A>G), and g.−2289 (G>A) significantly affected the transcription activity of the chicken *FGF9* gene. These DNA markers could be used in chicken breeding to improve egg-laying traits. In other animal species, no SNPs were reported in the promoter region of the *FGF9* gene.

## 5. Conclusions

In the process of chicken ovarian follicle development, the expression of *FGF9* mRNA shows significant differences before and after follicle selection. It is regulated by hormones such as FSH, estradiol, and progesterone, and may participate in the mechanism of follicle selection by modulating progesterone production. In the promoter region of *FGF9*, six SNP sites were identified, five of which were significantly associated with chicken egg-laying traits. The polymorphic sites g.−3906 (G>A), g.−3669 (A>G), and g.−2289 (G>A) significantly influence the transcription activity of the chicken *FGF9* gene. 

## Figures and Tables

**Table 1:** Primers used in this study.

Primer Name	Primer Sequence (5′-3′)	Annealing Temperature (°C)
FGF9-F	ACCTGTACCAGTCAAAGCAGAATA	62.0
FGF9-R	GTGCCAAACCCAGGAGATATAAGA	
GAPDH-F	GAGGGTAGTGAAGGCTGCTG	62.0
GAPDH-R	CACAACACGGTTGCTGTATC	
F1-KpnI	CGGGGTACCCAGTTTGCTGACTCAAGGC	61.0
R-MluI	CGACGCGTTCCGTAGTTTCTCCATTTGG	
F2-KpnI	CGGGGTACCTTCCTGCCTCTGTGTCTTG	58.4
R-MluI	CGACGCGTTCCGTAGTTTCTCCATTTGG	
F3-KpnI	CGGGGTACCCACAAGCCTTCACTCCTCA	58.4
R-MluI	CGACGCGTTCCGTAGTTTCTCCATTTGG	
F4-KpnI	CGGGGTACCTGCTTCTGGTGGGATTGT	58.4
R-MluI	CGACGCGTTCCGTAGTTTCTCCATTTGG	
F5-KpnI	CGGGGTACCCCCTTCTGTCCCTTTGCTAA	58.4
R-MluI	CGACGCGTTCCGTAGTTTCTCCATTTGG	
F6-KpnI	CGGGGTACCGCTGGAAGCGGTTCTGTAT	58.4
R-MluI	CGACGCGTTCCGTAGTTTCTCCATTTGG	
Mut-F-1965	TGAGGCTGCTGGCTCAGTGGTGGGGAAGCCC	65.0
Mut-R-1965	GGGCTTCCCCACCACTGAGCCAGCAGCCTCA	
Mut-F-2177	TGCAGGATGGGCAGCATGGGACCCTGCCCTG	65.0
Mut-R-2177	CAGGGCAGGGTCCCATGCTGCCCATCCTGCA	
Mut-F-2289	ATATATATGGCAAATACCGTGGAACATGACC	55.9
Mut-R-2289	GGTCATGTTCCACGGTATTTGCCATATATAT	
Mut-F-3669	TTCTTATTAATCTCAGTGGTTTTGCAGAGTA	55.9
Mut-R-3669	TACTCTGCAAAACCACTGAGATTAATAAGAA	
Mut-F-3906	AGCTCTCATTTACTTATACAAATCTTCCAGT	55.9
Mut-R-3906	ACTGGAAGATTTGTATAAGTAAATGAGAGCT	
P-FGF9-F1	CAGTTTGCTGACTCAAGGC	58.4
P-FGF9-R1	AGATTCACACCCACTTCCC	
P-FGF9-F2	AACTTCCTTCTGCCGCACA	58.4
P-FGF9-R2	CGGTGCCAATGATGATGT	

**Table 2:** Association of SNPs genotypes in promoter region of *FGF9* with laying traits of Jining Bairi hens.

Site	Traits	Genotype (Number of Hens)			*p* Value
g.-1965 G>A		*GG* (290)	*GA* (20)	*AA* (13)	
	AFE	150.18 ± 0.48	148.70 ± 1.83	148.46 ± 2.27	0.579
	E52	143.80 ± 1.34	151.75 ± 5.08	144.39 ± 6.31	0.320
	LCS	20.60 ± 0.96	26.10 ± 3.66	16.15 ± 4.54	0.205
	BW	1473.32 ± 9.89	1444.00 ± 37.67	1339.15 ± 46.73	0.017 *
	EW	33.16 ± 0.24	32.10 ± 0.91	31.49 ± 1.13	0.204
g.-2177 G>A		*GG* (296)	*GA* (31)	*AA* (12)	
	AFE	149.78 ± 0.48	148.74 ± 1.47	147.17 ± 2.37	0.465
	E52	143.85 ± 1.33	150.39 ± 4.12	136.83 ± 6.62	0.170
	LCS	20.51 ± 0.95	28.90 ± 2.94	13.33 ± 4.72	0.007 *
	BW	1470.97 ± 9.47	1428.55 ± 29.26	1415.00 ± 47.03	0.216
	EW	33.03 ± 0.23	33.37 ± 0.72	34.72 ± 1.16	0.342
g.-2289 G>A		*GG* (327)	*GA* (9)	*AA* (12)	
	AFE	149.89 ± 0.44	143.11 ± 2.67	150.42 ± 2.32	0.043 *
	E52	144.52 ± 1.26	149.44 ± 7.60	141.00 ± 6.59	0.703
	LCS	20.91 ± 0.90	22.33 ± 5.43	18.75 ± 4.70	0.870
	BW	1466.54 ± 9.37	1477.78 ± 56.49	1482.08 ± 48.92	0.936
	EW	32.96 ± 0.22	34.31 ± 1.30	33.51 ± 1.13	0.534
g.-3669 A>G		*AA* (299)	*AG* (31)	*GG* (11)	
	AFE	149.96 ± 0.48	147.84 ± 1.48	152.46 ± 2.49	0.226
	E52	144.36 ± 1.32	153.97 ± 4.11	137.27 ± 6.89	0.045 *
	LCS	21.00 ± 0.94	21.71 ± 2.93	20.91 ± 4.91	0.973
	BW	1466.83 ± 9.70	1470.00 ± 30.12	1456.36 ± 50.56	0.973
	EW	33.11 ± 0.24	33.02 ± 0.73	32.75 ± 1.23	0.954
g.-3770 A>G		*AA* (242)	*AG* (42)	*GG* (42)	
	AFE	149.87 ± 0.54	149.07 ± 1.29	150.67 ± 1.29	0.683
	E52	145.24 ± 1.49	141.91 ± 3.58	140.98 ± 3.58	0.427
	LCS	22.07 ± 1.06	17.02 ± 2.54	20.55 ± 2.54	0.180
	BW	1475.68 ± 10.78	1436.98 ± 25.88	1459.55 ± 25.88	0.361
	EW	32.88 ± 0.25	33.35 ± 0.60	33.03 ± 0.60	0.756
g.-3906 G>A		*GG* (323)	*GA* (22)	*AA* (12)	
	AFE	150.03 ± 0.45	147.46 ± 1.73	149.92 ± 2.34	0.354
	E52	144.10 ± 1.26	157.96 ± 4.82	140.92 ± 6.53	0.018 *
	LCS	20.77 ± 0.91	27.00 ± 3.50	18.67 ± 4.74	0.200
	BW	1465.44 ± 9.26	1515.23 ± 35.50	1451.67 ± 48.07	0.376
	EW	32.96 ± 0.22	33.78 ± 0.84	32.15 ± 1.14	0.486

Note: AFE—age at first egg; BW—body weight at first laying; E52—number of eggs at 52 weeks; EW—egg weight at first laying; LCS—largest clutch size. *p*-value indicates significant difference. When *p* < 0.05, the association between each genotype and the egg-laying trait is significantly different. Means ± SD were calculated for each phenotype. The * in the table indicates significant differences, *p* < 0.05.

## Data Availability

The original contributions presented in the study are included in the article, further inquiries can be directed to the corresponding author.

## Abbreviations

The abbreviations used in this manuscript are listed below:

AFE	Age at first egg
BW	Body weight at first laying
E2	Estradiol
E52	Number of eggs at 52 weeks
EW	Egg weight at first laying
FGF9	Fibroblast growth factor 9
FSH	Follicle Stimulating Hormone
IGF	Insulin-like growth factor
LCS	Largest clutch size
LW	Large white follicles
LY	Large yellow follicles
P4	Progesterone
Post-GCs	Granulosa cells of hierarchical follicles
Post-TCs	Theca cells of hierarchical follicles
Pre-GCs	Granulosa cells of pre-hierarchical follicles
Pre-TCs	Theca cells of pre-hierarchical follicles
SNP	Single-nucleotide polymorphism
SW	Small white follicle
SY	Small yellow follicle
